# Cases from Practice, with Remarks

**Published:** 1882-09

**Authors:** C. Pearson

**Affiliations:** Washington, D. C.


### CASES FROM PRACTICE, WITH REMARKS.

C. Pearson, M. D., Washington, D. C.

Mrs. H., aged 72, large fungus excrescence on neck, back of the left ear, the growth of three or four years. First saw her Jan. 6th, at which time it measured one and one-half inches in diameter, and about the same from base to top, very tender and vascular, bleeding from slightest touch. It had been pronounced by the members of the scientific (!) school, who had examined it, *Fungus Hœmatodes*, for which, of course, the knife was the only remedy, and $25 the lowest price (dirt cheap).

Having once cured a growth very similar in appearance on a lady about the same age, with Phos.$^{cm}$, I gave two powders of this, and repeated it again in seven days; but in two weeks, as there was no visible change, except perhaps a slightly less tendency to bleed, I concluded I had made a mistake, and gave two powders, six hours between, of the 10 M of Thuj., not having it higher, and repeated the two powders in the same way every seventh day for sixty days, or until the 24th of March, by which time one-half the tumor had sloughed off, and the balance was less sensitive and shrinking rapidly. After this no more medicine was given, and in four weeks more no part of the growth was visible, and only a small cicatrice remained to mark the spot. No external application of any kind was used, not even water, as I directed that the parts be kept perfectly dry. The old lady declared that she would willingly have paid $500 to have had it cured without cutting, and yet the expense did not exceed $12. As old *scientific* had *unanimously* decided that it could not be cured without the knife, he had quite a hunt for it after it had disappeared, but whether he concluded he was hunting on the wrong woman, or that he had never seen a tumor on her neck at all, I do not know, but he seemed to lose all interest in the search when he learned that it had been removed with nothing but powders taken internally.

CASE II. Miss C., aged 17, thin, delicate girl, had intermittent fever in the fall of 1881, of which she was cured (!) with *homœopathic Quinine* (whatever that is). Chills returned June 1st, 1882, and after having had two or three, tertian type, she applied to me. The indications for the remedy, as is so often the case in this disease, were far from being well marked. Chill first came at 11 A. M. Anticipating one hour, third attack at 8, lasted one hour, little or no nausea, no great thirst or pain anywhere, fever for two or more hours, with thirst and headache, then perspiration without any marked symptoms. On the day preceding the chill, I gave four powders of Aranea diad.$^{cm}$, three hours between, the next morning chill came two hours earlier, and much more severe, going off by vomiting, high fever and of longer duration, altogether the worst day she had had. Sac. lac., no more chills and no fever since.

Now, in reference to the former case, would not the disciple of Hippocrates, the modern self-styled *rational* homœopath, have been equally as incredulous as his twin brother, the allopath, and as much disposed to use the knife, particularly if he should have a great desire to play surgeon, and to always be on the lookout for something to cut. To the mechanical part of surgery I have no objections, whatever; it is a business that has no necessary connection with medicine, but is, and should be considered, as distinct from it as is dentistry. But I have a supreme contempt for the specialist, who sees in every abnormal growth, whether of the eye, ear or any other part, something requiring an operation, and at which he proceeds to poke, and scrape, and cut; and then to syringe, and salve, and cerate, until, if he succeeds in healing up the sore he has made, he has done a big thing. I have no patience with specialists; if a physician is competent to practice medicine at all, he should be as familiar with one part of the body as another. The gynecologist finds nine-tenths of all female complaints located in the uterus, and hence, conceives the necessity of getting inside of his patient with a light, to find the cause of the mischief, and should he discover an *effect*, he is all right, for he is death on effects; and just so it is with the eye and ear men, the lung doctor, the cancer doctor, the worm, the corn and the pile doctor, and in this particular, as in many other respects, our school is aping the empiricism of the other. Why will not physicians learn to *cure* their patients, and let effects take care of themselves? But no! This would not be progressive *rational* Homœopathy. If my allopathic neighbor has a microscope, laryngoscope, speculum, pessary, forceps, probe and hypodermic syringe, I must be in style and get the same, and though I may never be able to cure any one by their aid, that could not have been cured much quicker and better without, I may at least succeed in scaring somebody and getting the reputation of being a wonderful doctor, in the same sense that Dr. Valentine viewed his preacher. The doctor said their minister was a terrible powerful preacher; that he had only preached for them six months, till he had knocked two pulpits to pieces and clanged the insides out of three Bibles; he was an awful preacher. And this "doing something" seems to satisfy the people, whether it be of any utility or not. But the question is, are these whims of the patients to be humored and the physician's judgment to be biased thereby? If a child cries for candy, must the mother give it, without regard to consequences?

There are currents, or floods in the mental world as well as in the physical; certain drifts, on which and with which some physicians delight to float into notoriety. At the present time this drift is liberalism. The allopathic fraternity, for the past fifty years, having failed to crush out Homœopathy by their opposition, have all at once become very liberal, and like the spider to the fly, invite us into their parlors, where they, obviously, intend to drug us to death, and the unwary fly indirectly accepts the invitation, as the following resolution adopted at the last meeting of the American Institute will show:

"*Resolved*, That it is the sense of the American Institute that no physician can properly sustain the responsibilities, or fulfill all the duties of his professional relations, unless he enjoys absolute freedom of medical opinion, and unrestricted liberty of professional action, as provided for in the Code of Ethics of this Institute."

One member made a few remarks in opposition, which caused the president to remark that he regretted that the vote was not unanimous. Come to my arms, my long lost brother! And if you don't like this hateful word Homœopathy, we will drop it next year. Anything for harmony.

